# The procrastination trap: How temperament undermines students’ career readiness in Poland

**DOI:** 10.1371/journal.pone.0358981

**Published:** 2026-10-16

**Authors:** Klaudia Martynowska, Piotr Mamcarz, Ewa Domagała-Zyśk, Alessandro Lo Presti, Andrew Rothwell

**Affiliations:** 1 Department of Special Pedagogy, John Paul II Catholic University of Lublin, Lublin, Poland; 2 Department of Psychology, John Paul II Catholic University of Lublin, Lublin, Poland; 3 Department of Psychology, Università degli Studi della Campania “Luigi Vanvitelli”, Caserta, Italy; 4 School of Business and Economics, Loughborough University, Leicestershire, United Kingdom; University of Padova, ITALY

## Abstract

This study examines links between temperament, procrastination tendencies, and self-perceived employability among Polish university students, addressing a notable gap in research on behavioral postponement effects in European higher education contexts. Cross-sectional survey data were collected from 522 first-year undergraduates enrolled in 16 fields of study at a mid-sized Polish university. Temperament was assessed with the Formal Characteristics of Behaviour – Temperament Inventory, Revised (FCB-TI(R)); procrastination with the Polish version of Lay’s General Procrastination Scale, scored as a single general index; and self-perceived employability with the Polish Self-Perceived Employability Scale (internal and external subscales). Grade point average (GPA) was obtained from university records. Bootstrapped regression-based models estimated the indirect association between each of the seven temperament dimensions and internal employability through procrastination. Procrastination was associated with lower briskness, rhythmicity, sensory sensitivity, endurance and activity, and with higher emotional reactivity and perseveration. For all seven dimensions the indirect association with internal employability through procrastination was significant, with completely standardised indirect effects between  .02 and  .05; for endurance and rhythmicity the association was indirect only. Procrastination was also associated with lower GPA (r = −.17) and with lower external employability (r = −.18), whereas GPA was unrelated to any employability index. Because the design is cross-sectional and all predictors were measured concurrently, these findings describe patterns of association that are consistent with, but do not establish, a mediating process. The results identify temperamentally patterned procrastination as a modifiable correlate of students’ career confidence, and caution against treating academic grades as a proxy for employability readiness.

## Introduction

Poland shares many employment concerns with its European neighbors, yet graduate employability in the country is relatively under-researched. The Polish higher education sector grew rapidly from 404,000 students in 1990/1991 to 1.9 million in 2010 [1]. However, this was followed by a subsequent decline to reach just over 1.2 million in 2021, principally due to demographic factors, like lowering birth rate in the last three decades or extensive emigration waves of secondary school graduates [2]. Moreover, it has been suggested that the earlier expansion did not meet the labor market needs [3], suggesting that while individuals attain degrees, consistent with other European countries [4], it may not be in fields desired by the industry or service sector [5]. In Poland, this phenomenon may disguise the true picture of employment, as while the data suggest that in 2020 only 3.5% of the registered unemployed were graduates, this may be due to that graduates are choosing under-employment [6] over unemployment. Poland is one of seven European countries where this trend is observed [7]. This is a matter of concern since over-qualified workers in Poland typically earn 25% less than those who are well-matched [3]. For some time, these phenomena had stimulated debate in Poland on the quality of higher education and employability of graduates, leading to the establishment of the National Information Processing Institute’s Polish Graduate Tracking System or ELA (Survey of the Economic Fate of Graduates) in 2015.

Employment transitions for graduates in Poland are a source of increasing scrutiny for academics and policymakers. For example, recent publications have confirmed labor market disparities among engineers, computer scientists, and social scientists [8]. Employers in Poland value both technical and soft skills such as teamwork [9]. Education is often a signal of not only a high level of human capital but also of social capital [10]. Other recent Polish studies have focused on **c**ompetences in university-to-work transition in a Polish context [11] such as development of professional skills [12], career procrastination in university-to-work transition [13], and employability and networking competence [14].

As teachers we have been concerned about the lack of apparent urgency in improving employability outcomes. Hence, a substantial motivation for this research was to provide evidence for developing policy and curriculum interventions that might better serve students’ needs. At the same time, we are keen to avoid the socially constructed and stereotypical assumptions often found in “generational” research [2,15]. Following Rudolph et al., we adopted a “lifespan” perspective that considers human development as a process throughout life influenced by various factors that predict developmental outcomes [15]. Here, development is the product of individual decisions and actions that may be affected by context and circumstances. For many contemporary HEIs, full-time graduate employment is a key performance indicator [16], preferably achieved within three or six months of graduation. However, graduation and employment are not directly connected; one can graduate from a prestigious faculty and face problems getting employment relevant to the subject area. For individual students, self-perceived employability [17], defined as the potential ability to attain employment appropriate to one’s qualification level might serve as a prominent personal resource for facing the transition to work, which could also be achieved within the higher education environment, before the actual transition to work.

The upskilling thesis [18] suggests that the contemporary work world requires a higher level of education, training, and experience to obtain a job that matches graduate-level expectations [19,20]. At the same time, in a world characterized by increasing uncertainty, does stress have a deleterious impact on students, to the extent that they fail to engage with their studies or procrastinate in seeking employment opportunities?

As it was justified above, employability should be considered as a personal quality which immensely supports occupational perspectives and well-being of young adults. The question arises what are its correlates and ways to boost its quality? In this paper we propose to examine links between self-perceived employability and two other personal features: temperament and procrastination tendencies.

### Temperament

Temperament is relatively stable across one’s lifespan and is relevant to this study in the context of reactions and behaviors resulting from energetic and temporal characteristics [21].

Temperament refers to biologically grounded individual differences in the formal – energetic and temporal characteristics of behaviour, that is, in how rather than why or what a person acts [22]. Its relative stability is a matter of degree rather than of kind: meta-analytic evidence from longitudinal studies indicates substantial rank-order consistency of dispositional traits, which increases with age but never approaches unity, and which coexists with systematic mean-level change [23]. We therefore treat temperament as a comparatively stable but not immutable disposition.

Temperament can be modulated by environmental factors — particularly stress or excessive demands placed on an individual [22]. A widely used framework for describing this construct is Strelau’s Regulative Theory of Temperament (RTT) [24], which organizes temperament into two broad clusters of traits: those related to reactivity and those related to activity.

Reactivity traits describe the intensity of an individual’s response to stimulation. This cluster includes sensory and emotional sensitivity, and endurance – a person’s capacity to sustain effort or work under demanding conditions. Strelau and Zawadzki (2011) linked this reactivity dimension to fearfulness, suggesting that individuals higher in reactivity may be more prone to fear responses [25].

Activity traits, by contrast, describe the amount and pattern of goal-directed behavior a person engages in, including whether that behavior tends to seek out or avoid environmental stimulation. More recently, researchers have proposed adding an eighth (or, depending on the formulation, additional) dimension: rhythmicity which captures how consistently or predictably an individual responds to novel stimul [26].

A central postulate of RTT is regulatory: temperament traits determine the stimulative value that a given activity has for a person, and individuals organise their behaviour so as to keep stimulation within a preferred range [22]. This postulate matters for the present study because it implies that temperament should shape outcomes such as career confidence indirectly – through the habitual patterns of action, delay and avoidance by which people regulate the stimulation that tasks impose on them – rather than directly.

Given that temperament shapes how individuals appraise and respond to demanding or uncertain situations, it is plausible that these same dispositional traits also influence how confidently a person judges their own prospects in the labor market — an outcome commonly studied as self-perceived employability. The following section reviews this emerging line of research.

### Procrastination

From the environment to health, it is common for individuals to avoid dealing with concerns and let them deepen over time [27,28] to the extent that procrastination became a key public concern, as it leads to negative outcomes in health behavior [29]. Procrastination occurs when there is a significant time gap between the intention to undertake an activity and actually doing it. It has been linked to agitation and other variables such as low self-control, rebellion, and extraversion [30]. The most comprehensive quantitative synthesis to date frames procrastination as a quintessential failure of self-regulation, with task aversiveness, impulsiveness, low conscientiousness and low self-efficacy as its most robust correlates [31].

When it comes to universities or other higher education institutions (referred to as: HE context), a recent study revealed that approximately 46% of undergraduate and 60% of graduate students regularly postpone academic tasks [32]. Procrastination has been consistently shown to have negative effects on students’ learning approaches [33] and academic performance [34,35], psychological well-being [36], and stress [37]. In particular, first-year undergraduates (the focus of our study) struggle to manage their time effectively and tend to procrastinate because of overestimating their academic abilities [38]. They are particularly susceptible to academic procrastination as they encounter various novel opportunities (e.g., socialization, volunteering, and athletics) and challenges (e.g., academic setbacks and decisions and greater autonomy) during the transition to postsecondary education.

Longitudinal evidence has begun to establish that these costs extend beyond the academic domain: in a large Swedish cohort, higher procrastination predicted subsequent mental-health problems, disabling pain, poor sleep, physical inactivity, loneliness and economic difficulty nine months later [39], and a longitudinal test in adults identified stress and health behaviours as the pathways involved [40].

Our study explored procrastination as a voluntary delay in an intended course of action despite the awareness of its adverse effects [41]. We are particularly interested in how this cognitive failure transcends to job-seeking activity of graduates. In the context of graduates’ future employability, previous study showed that high levels of procrastination toward job-seeking may lead to an increase in perceived hopelessness [42]. Beyond the student years, procrastination has been associated with lower salaries, shorter employment duration and a greater likelihood of unemployment in a large adult sample, and procrastinators tend to hold jobs that are lower in intrinsically rewarding qualities [43]. Identification with a procrastinator identity has likewise been linked to career and financial regret [44], and to weaker professional identity through reduced career decision-making self-efficacy [45].Thus, it is of crucial importance to investigate factors affecting relation of procrastination and perceived employability.

### Employability

Employability can be examined from both social and individual perspectives. The social perspective is connected with the labor market (the higher the level of employability, the better the health of the economy), recruitment strategies, vocational counseling for the unemployed or underemployed, and development and policy strategies of state and private institutions [46]. This can also be extended to strategies in HEIs to develop career readiness [47] or encourage students to acquire work experience through internships or placements [48]. The individual perspective considers several individual factors that construct a person’s employability, such as subject knowledge, job experience, especially when reflected upon [49], and soft skills, such as self-confidence, self-awareness, communication, efficacy beliefs, metacognition, and enterprise skills. The individual perspective can also embrace the self-perception of internal and external employability as separate factors. The former includes a self-evaluation of skills and attributes as well as awareness of opportunities. The latter includes the perceived desirability of the subject area and the state of the external labor market [17,50]. Procrastination and temperamental features can be regarded as internal factors, and thus far, to our knowledge, have not been studied in the context of self-perception of employability. Hence, in our study, employability is seen as a multidimensional construct related to deploying one’s abilities, skills, beliefs, and knowledge to maintain meaningful employment that is relevant to one’s qualification and aspirations [50,51].

Dispositional antecedents of self-perceived employability have been documented across cultures. In a three-country study, the activity and neuroticism dimensions of normal personality predicted perceived employability, with these relations fully or partially mediated by proactive attitude [52], and in a Polish student sample the effects of conscientiousness, emotional stability and extraversion on self-perceived employability operated largely through hope and grit [53]. What these studies share is a two-stage structure: stable dispositions are related to employability appraisals not directly but through the habitual, motivationally loaded behaviour they generate. Procrastination and temperamental characteristics can both be regarded as internal factors and, to our knowledge, have not previously been examined together in relation to self-perceived employability.

### The present study: Rationale and hypotheses

Three considerations motivated the decision to model procrastination as the intervening variable between temperament and self-perceived employability rather than the reverse, or a simple set of bivariate associations.

RTT treats temperament as a distal, biologically anchored regulator of the stimulative value of activity [24]. Temperament is not a belief about the labour market and contains no career-relevant content; it therefore has no plausible direct semantic route to a judgement about one’s employment prospects. What it does supply is a characteristic manner of engaging with demanding tasks. Procrastination is precisely such a manner of engaging – a behavioural, proximal expression of self-regulation under stimulation [31,41] - and is thus the natural candidate to carry temperamental variance forward into evaluative outcomes.

The temperament-to-procrastination link has been demonstrated in a Polish adult sample using the same instrument employed here: emotional reactivity and perseveration correlated positively with procrastination, whereas briskness, endurance and rhythmicity correlated negatively, and emotional reactivity mediated the effect of affective temperament on delay [54]. Related work using different trait models converges on the same ordering, with impulsivity and behavioural-inhibition traits predicting passive procrastination through mind-wandering [55], and with dispositional traits predicting procrastination through emotional intelligence and self-efficacy. The procrastination-to-employability link is supported independently by evidence connecting delay with career-related hopelessness [42], procrastinator identity with career regret [44], and procrastination with professional identity through career decision-making self-efficacy [45]. Developmentally, temperamental self-regulation and negative affectivity are already associated with delay behaviour in pre-school children [56], long before any labour market appraisal is possible. No study has yet joined the two links in a single model, which is the gap the present study addresses.

Self-perceived employability is an efficacy-type appraisal, and enactive mastery – the accumulated record of one’s own task completion – is the strongest source of efficacy beliefs [57]. Habitual delay degrades exactly that record. A student who repeatedly submits work late, or postpones the search for placements, accumulates evidence of unreliability that is available to them when they judge whether they could obtain a suitable job. This is why we expected procrastination to be linked more strongly to *internal* employability - a judgement about one’s own skills and attributes – than to *external* employability, which concerns the state of the labour market and is less informed by personal task history.

We emphasise at the outset that this reasoning specifies a plausible ordering; it does not licence causal language. Our data are cross-sectional and all three constructs were measured at a single occasion. A regression-based mediation model of such data yields an unbiased estimate of the indirect effect only if (i) the causal order X → M → Y is correct, (ii) no unmeasured variable confounds the X–M, X–Y or M–Y relations, and (iii) the relations are correctly specified and free of interference [5,58]. None of these assumptions was tested here, and assumption (ii) in particular is implausible in a single-occasion self-report design. Accordingly, we describe all findings in terms of association and *indirect association*, and reserve the term “mediation” for the statistical model rather than the process.

We state hypotheses about the component associations first, and only then the indirect association that presupposes them.

**H1a.** Temperament dimensions indexing behavioural drive and temporal regularity — briskness, activity, endurance and rhythmicity — will be negatively associated with procrastination, whereas emotional reactivity and perseveration will be positively associated with it. No directional prediction is made for sensory sensitivity.

**H1b.** Procrastination will be negatively associated with self-perceived internal employability.

**H1c.** Temperament dimensions will be associated with self-perceived internal employability, in the directions implied by H1a and H1b.

**H2.** For each temperament dimension, the association with self-perceived internal employability will include a significant indirect component through procrastination (that is, the product *ab* will differ from zero), consistent with — though not demonstrating — an intervening role of procrastination.

Two questions were treated as exploratory rather than hypothesised. **RQ1:** how do temperament and procrastination relate to an objective indicator of academic performance (GPA), and is GPA in turn related to employability appraisals? **RQ2:** does the pattern observed for internal employability extend to external employability, which by definition concerns the labour market rather than the self?

We deliberately do not hypothesise an indirect effect of *temperament as a single predictor*. The FCB-TI(R) yields no total score, its dimensions are not all positively keyed with respect to any single latent continuum, and – as reported below – a principal component analysis of the seven scale scores in this sample yielded three components with eigenvalues above unity, the first accounting for only 31.7% of the variance. No defensible composite exists, so the model is specified at the level of the seven dimensions (Fig 1).

**Fig 1:**
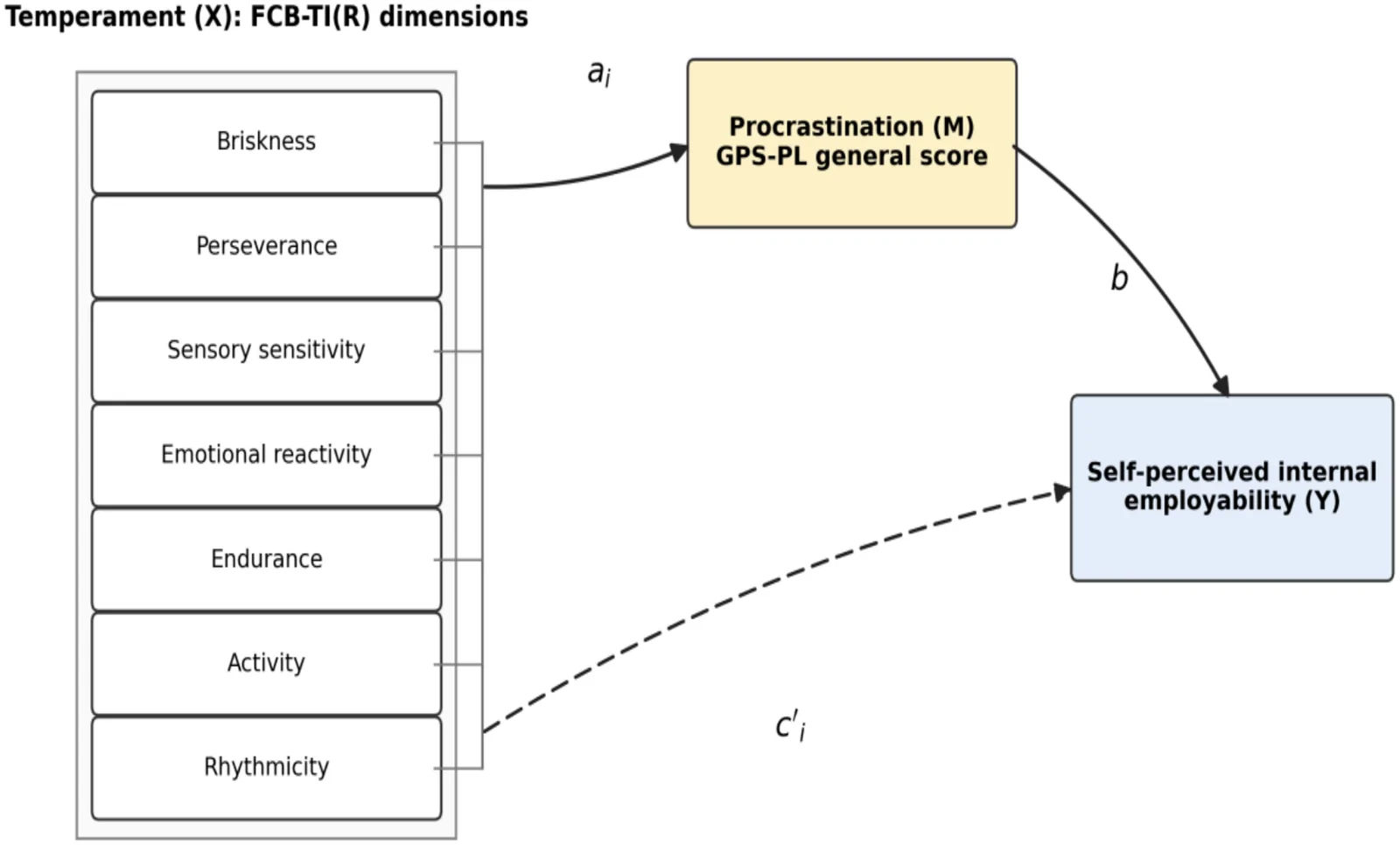
Conceptual model. Seven separate models for mediation were estimated, one per temperament dimension: *a*_*i*_ *=* effect of temperament dimension *i* on procrastination; *b* = effect of procrastination on internal employability, adjusting for temperament; *c*^*′*^_*i*_  *=* direct effect of dimension *i,* adjusting for procrastination; *a*_*i*_*b =* indirect effect.

Conceptual model of the associations was examined. Seven separate models were estimated, one for each FCB-TI(R) dimension. Paths are labelled following the notation used in Table 4. The model is descriptive of a hypothesised ordering; the cross-sectional design does not permit causal identification.

## Data and methods

### Participants

The study was conducted at a mid-sized university in Poland among first-year undergraduate students across different faculties. A total of 600 first-year students voluntarily participated in the study. After removal of records with incomplete responses and invariant response patterns (see Procedure), the final analytic sample comprised 522 students (86.9% of those who began the survey), enrolled in 16 fields of study. Of these, 373 (71.5%) were women and 149 (28.5%) were men; mean age was 19.91 years (SD = 2.40, range 17–57). Demographic characteristics are reported in Table 1.

**Table 1:** Participant demographic characteristics (N = 522).

Characteristic	Value
Age (years)	M = 19.91 (SD = 2.40); range 17–57
Gender: female	373 (71.5%)
Gender: male	149 (28.5%)
Fields of study represented	16
Employment: full-time	38 (7.3%)
Employment: part-time	26 (5.0%)
Employment: odd jobs	85 (16.3%)
Employment: not employed	373 (71.5%)
Residence: < 20 K inhabitants	275 (52.7%)
Residence: 20 K – 49 K	58 (11.1%)
Residence: 50 K – 100 K	58 (11.1%)
Residence: > 100 K	131 (25.1%)
Financially independent: yes	74 (14.2%)
Financially independent: no	448 (85.8%)
Accommodation: family home	127 (24.3%)
Accommodation: rented room	311 (59.6%)
Accommodation: hall of residence	75 (14.4%)
Accommodation: own apartment	6 (1.1%)

Counts may not sum to 522 because of occasional item non-response (three participants did not report accommodation type). Percentages are computed on valid responses

### Procedure

Before data collection, approval from the Ethics Committee of the Institute of Pedagogy Institute John Paul II Catholic University of Lublin (no. 2020-10-06/1) was obtained. Data were collected via an online between 01.06.2022 and 30.06.2022. The questionnaire battery was programmed and administered as a single digital form on the LimeSurvey platform; the instruments were reproduced in digital form under the terms of the relevant copyright permissions. Students were invited by email and through faculty announcements and completed the questionnaire on that platform; participation was voluntary and unremunerated. The research followed the ethical rules of the American Psychological Association and was prepared in accordance with the Declaration of Helsinki. All subjects gave their informed consent for inclusion before they participated in the study. Consent was provided electronically: each participant was presented with a clear description of the study’s aims, voluntary nature, and right to withdraw at any time, and was required to actively confirm agreement by selecting “Yes” to the participation question before proceeding to the questionnaire. This digital confirmation served as documented written consent. No verbal consent was used. The consent procedure was approved as part of the ethical review. The researchers where not using any personal information of participants and were working on anonymized dataset. Data quality was screened before analysis using three criteria applied in the order given: (i) completeness — records with missing responses on any scale item were excluded; (ii) invariant responding — records showing a single response option used throughout an entire instrument were excluded; and (iii) implausible response duration. No dedicated attention-check or instructed-response items were embedded in the questionnaire; this is a limitation of the design and is discussed below. Item-level missingness among retained records was below 1%. One retained record contains a single unanswered item on the Activity subscale, and the subscale score stored for that case cannot be reconstructed from its item responses; the case was retained as recorded, and sensitivity analyses in which the score was prorated from the answered items, and in which the case was excluded, changed no coefficient reported in Table 3 or Table 4 by more than  .003. Grade point average could not be retrieved for 71 of the 522 retained participants (13.6%), mainly because the students concerned had not yet completed both assessed semesters at the time of extraction; analyses involving GPA are therefore based on n = 451 and were computed with pairwise deletion. Participants with and without an available GPA did not differ significantly on procrastination, temperament or employability.

### Measurements

#### Procrastination.

Procrastination was measured with the Polish-language version of Lay’s General Procrastination Scale [59], which in the form used here comprises 15 items rated on a five-point scale from 1 (*never*) to 5 (*always*), yielding a possible range of 15–75. The scale measures the global tendency to postpone intended action across everyday and academic tasks. In Lay’s original work [59], factor analysis returned two components — one comprising items describing *dilatory behaviour and last-minute completion*, the other items describing *promptness and timely task completion*, which are reverse-keyed — with internal consistencies of α = .85 and α = .75 and a general-score α of  .84; because the content of the two components differs only in item keying rather than in substance, Lay concluded that the instrument is functionally unidimensional. We therefore used a single general score throughout, and the abstract has been revised so that it no longer implies that academic and decisional subtypes of delay were distinguished.

The Polish items were prepared for the present programme of research using a forward- and back-translation procedure. The English source items [59] were translated into Polish, and the Polish version was then independently back-translated into English by a bilingual translator who had not seen the original instrument. The back-translation was compared item by item with Lay’s original wording, and the small number of discrepancies identified — all concerning idiomatic rather than substantive content — were resolved by discussion within the research team before the Polish wording was fixed. The resulting version has not previously been published with full psychometric documentation, which we regard as a limitation, and we therefore report its properties in the present sample in full. In the present sample the 15-item general score showed acceptable internal consistency (α = .80, ω = .80, mean inter-item r = .21). During re-analysis for this revision we identified a scoring error in the original data file: one reverse-keyed item (“I usually accomplish all the things I plan to do in a day”) had not been recoded, and its corrected item–total correlation was − .47. After correct recoding, the item–total correlation was + .47 and α rose from  .73 to  .80. All results reported below use the corrected scoring. Results obtained with the original scoring are substantively identical in sign, significance and interpretation for every analysis; the largest change in any correlation coefficient was  .04.

#### Self-perceived employability.

The Polish Self-Perceived Employability Scale used here was developed from the 12-item instrument of Rothwell and colleagues [17] and has been employed previously with Polish first-year undergraduates [53]. Items are rated from 1 (*strongly disagree*) to 5 (*strongly agree*).

The structure of the Polish instrument is set out here in full. The Polish version retains **six** of the twelve original items. Principal component analysis with varimax rotation and Kaiser normalisation was well supported (KMO = .74; Bartlett’s test of sphericity χ^2^(15) = 973.0, *p* < .001) and yielded two components with eigenvalues above unity (2.83 and 1.30) accounting jointly for 68.8% of the variance. Four items loaded on an *external* employability component: “My chosen field of study is regarded as prestigious” (.79), “Graduates from my field of study are in demand on the labour market” (.78), “Completing a degree in my field is seen as a route to success” (.83) and “University graduates in general are currently in demand” (.69); cross-loadings ranged from  .06 to  .21. Two items loaded on an *internal* employability component: “I am generally confident that I will do well in job interviews” (.89) and “I feel I could get any job so long as it is reasonably relevant to my skills” (.91); cross-loadings were  .18 and  .08. Communalities ranged from  .48 to  .84. Scores are sums, so the possible ranges are 4–20 (external), 2–10 (internal) and 6–30 (total), which is why the observed subscale means sum exactly to the general score. A parallel analysis over 500 simulated data sets retained the same two components, and a confirmatory factor analysis specified from this solution fitted the data well (χ^2^(8) = 9.29, p = .32, CFI = .999, TLI = .998, RMSEA = .018, SRMR = .018) and fitted substantially better than a one-factor alternative (Δχ^2^(1) = 239.8, p < .001; CFI = .751, RMSEA = .226); the latent correlation between the two facets was  .41.

Reliability is reported separately for each subscale. Internal employability: α = .79; because this subscale comprises only two items, a congeneric measurement model is under-identified and ω is not estimable, so the more informative indices are the inter-item correlation (r = .65) and the Spearman–Brown coefficient (.79). External employability: α = .79, ω = .79. Total scale: α = .77, ω = .78. The brevity of the internal subscale is a genuine limitation, since this variable is the outcome in every model reported below – yet is it clearly mentioned in the limitations section.

#### Temperament.

Temperament traits were assessed using the Formal Characteristics of Behavior-Temperament Inventory revised version (FCB–TI). This is a self-report scale designed by Cyniak–Cieciura et al.[26],to measure basic, biologically determined personality dimensions that describe formal aspects of behavior and comprises 100 items on a 4-point scale. The instrument was constructed in Polish, so no translation was required, The items form seven subscales: briskness, perseverance (temporal characteristics), sensory sensitivity, emotional reactivity, endurance, activity (energetic aspects of behavior), and rhythmicity. In the original validation, α coefficients and stability indices ranged from  .68 to  .93. In the present sample, subscale reliabilities ranged from α = .78 (sensory sensitivity) to α = .87 (emotional reactivity), with ω between  .78 and  .87.

#### Academic performance.

As an objective indicator of academic performance, grade point average (GPA) was obtained (with the consent of the participants) from the university database using secondary data. GPA was computed as the mean of the two first-year semester averages on the Polish 2–5 marking scale. We incorporated this measure in order to include at least one variable not derived from self-report. GPA is treated as an exploratory correlate (RQ1) and is not part of the hypothesised model in Fig 1; it is included because academic performance is the outcome most often assumed, in institutional practice, to index employability readiness, and because that assumption has rarely been tested directly.

The number of items, descriptive statistics and internal consistency of every scale *as estimated in the present* sample are presented in Table 2. Both Cronbach’s α and McDonald’s ω are given, because α is known to be sensitive to scale length and to tau-equivalence violations.

**Table 2:** Number of items, descriptive statistics and internal consistency of all measures in the present sample.

Measure (scale)	Items	M (SD)	Range	α	ω	Mean r
Procrastination (GPS-PL)	15	40.60 (9.01)	15–75	.80	.80	.21
Self-perceived employability — total	6	21.07 (3.73)	6–30	.77	.78	.36
Internal employability	2	7.06 (1.58)	2–10	.79	—	.65
External employability	4	14.01 (2.91)	4–20	.79	.79	.48
Briskness (FCB-TI(R))	15	42.36 (6.95)	15–60	.79	.79	.21
Perseveration (FCB-TI(R))	15	45.60 (6.85)	15–60	.81	.81	.22
Sensory sensitivity (FCB-TI(R))	15	43.90 (6.43)	15–60	.78	.78	.19
Emotional reactivity (FCB-TI(R))	15	42.41 (8.52)	15–60	.87	.87	.31
Endurance (FCB-TI(R))	15	33.05 (8.24)	15–60	.85	.85	.27
Activity (FCB-TI(R))	15	38.54 (7.61)	15–60	.83	.84	.24
Rhythmicity (FCB-TI(R))	10	20.06 (6.03)	10–40	.85	.86	.37
Grade point average	—	4.15 (0.39)	2–5	—	—	—

N = 522 for all scales; N = 451 for grade point average. α = Cronbach’s alpha; ω = McDonald’s omega; Mean r = mean inter-item correlation. Range = possible range of the summed score.

### Statistical analyses

All analyses were conducted in IBM SPSS Statistics 29 with the PROCESS macro (Model 4) and independently reproduced from the deposited data file in Python (NumPy 2.4, SciPy 1.17, statsmodels) and in R 4.3.3 (psych for reliability and component analyses, lavaan for the path models); the three implementations agreed to three decimal places. Continuous variables are summarised as means and standard deviations, categorical variables as counts and percentages. Zero-order associations were estimated with Pearson correlations. The significance level was set at *p* < .05, two-tailed. Because 66 correlations are reported in Table 3, we interpret only coefficients that are both statistically significant and of a magnitude consistent with the hypotheses, and we report effect sizes throughout rather than relying on significance alone.

**Table 3:** Intercorrelations among the study variables.

Variable	1	2	3	4	5	6	7	8	9	10	11
1. GPA	—										
2. Procrastination	−.17**	—									
3. Briskness	.02	−.32**	—								
4. Perseveration	.08	.09*	−.13**	—							
5. Sensory sensitivity	.00	−.15**	.20**	.23**	—						
6. Emotional reactivity	.04	.21**	−.28**	.62**	.10*	—					
7. Endurance	−.09*	−.15**	.24**	−.40**	−.05	−.49**	—				
8. Activity	−.17**	−.12**	.17**	−.04	.17**	−.07	.09*	—			
9. Rhythmicity	.07	−.26**	.06	−.25**	−.07	−.08	.06	.05	—		
10. External employability	.02	−.18**	.02	−.02	−.03	.07	−.06	.15**	.06	—	
11. Internal employability	.00	−.19**	.15**	−.11*	.15**	−.26**	.10*	.19**	.04	.32**	—
12. SPE general score	.01	−.22**	.08	−.06	.04	−.06	.00	.19**	.07	.92**	.67**

N = 522; N = 451 for correlations involving GPA (pairwise deletion). * *p* < .05, ** *p* < .01 (two-tailed). SPE = self-perceived employability. Means, standard deviations and reliabilities for these variables are given in Table 2.

The continuously measured variables were not dichotomised at any point. Dichotomising continuously measured variables discards variance, imposes sample-dependent cut points and is inconsistent with the continuous treatment of the same variables in the path models. All questions concerning group differences in procrastination, temperament and employability are therefore addressed with correlations and ordinary least squares regressions on the continuous scores, and effect sizes (*R*^2^, Cohen’s *f*^2^) are reported.

Indirect associations were estimated with regression-based path analysis using ordinary least squares, separately for each of the seven temperament dimensions. Path *a* is the regression of procrastination on temperament dimension *i*; path *b* is the partial regression of internal employability on procrastination, adjusting for temperament; *c* is the total association of temperament with internal employability; and *c′* is the direct association adjusting for procrastination. The indirect component is the product *ab*. Confidence intervals were obtained by bias-corrected and accelerated bootstrapping with 5,000 resamples [60], which does not require the sampling distribution of the product to be normal. All path coefficients are unstandardised; standardised coefficients and the completely standardised indirect effect (*ab* expressed in units of the outcome’s standard deviation) are reported as effect sizes.

We do not frame the analysis in terms of the causal steps of Baron and Kenny [61]. That framework requires a significant total effect before an indirect effect may be examined, a requirement that current methodological consensus rejects: a total effect can be non-significant while a genuine indirect component is present, particularly when direct and indirect components have opposing signs or when the total effect is small relative to sampling error [58,62,63]. One dimension in our data (rhythmicity) shows exactly this pattern. We therefore follow contemporary practice: the quantity of interest is the indirect effect itself, and we classify each result using the typology of Zhao, Lynch and Chen [62] (*complementary* when *ab* and *c′* share a sign; *indirect only* when *ab* is significant and *c′* is not). We stress that a confidence interval excluding zero establishes that the product of two regression coefficients is unlikely to be zero *within the assumed model*; it does not establish mediation in the causal sense [64].

Three sets of supplementary analyses were performed: (i) all path models were re-estimated with gender and age as covariates; (ii) the models were re-estimated with external employability and with the general employability score as alternative outcomes (RQ2); and (iii) Harman’s single-factor test was conducted on the scale scores as a coarse screen for common method variance [65].

## Results

### Zero-order correlations

For internal employability (two items) the inter-item correlation is r = .65 and the Spearman–Brown coefficient is  .79. GPS-PL = Polish version of the General Procrastination Scale; FCB-TI(R) = Formal Characteristics of Behaviour – Temperament Inventory, Revised. McDonald’s ω was estimated from a congeneric one-factor confirmatory model fitted by maximum likelihood; for internal employability, which comprises two items, that model is under-identified and ω is not reported. For the multi-item scales, ω-total from a Schmid–Leiman bifactor solution lies between  .81 and  .91.

Zero-order correlations are reported in Table 3. Procrastination was significantly correlated with every other variable in the matrix. Describing the temperament coefficients in absolute values, the associations between procrastination and the seven temperament dimensions ranged from |r| = .09 to |r| = .32, all *p* < .05. The signed coefficients were negative for briskness (r = −.32), rhythmicity (r = −.26), sensory sensitivity (r = −.15), endurance (r = −.15) and activity (r = −.12), and positive for emotional reactivity (r = .21) and perseveration (r = .09).

Procrastination correlated negatively with internal employability (r = −.19, *p* < .001), external employability (r = −.18, *p* < .001) and the general employability score (r = −.22, *p* < .001). Among the temperament dimensions, internal employability correlated positively with activity (r = .19), briskness (r = .15), sensory sensitivity (r = .15) and endurance (r = .10), and negatively with emotional reactivity (r = −.26) and perseveration (r = −.11); rhythmicity was not significantly correlated with internal employability (r = .04, *p* = .35). External employability, by contrast, correlated significantly with only one temperament dimension, activity (r = .15). GPA correlated negatively with procrastination (r = −.17), activity (r = −.17) and endurance (r = −.09), and was unrelated to perseveration (r = .08, *p* = .08) and to all three employability indices (|r| ≤ .02).

### Procrastination, academic performance and employability appraisals

Procrastination predicted GPA (b = −0.008, SE = 0.002, β = −.17, t(449) = −3.70, *p* < .001, R^2^ = .030, f^2^ = .031): a one standard deviation increase in procrastination corresponded to a decrease of approximately 0.07 grade points. Neither internal employability (β = −.00, t(449) = −0.03, *p* = .97) nor the general employability score (β = .01, t(449) = 0.26, *p* = .80) predicted GPA. The pattern is therefore that *procrastination was associated with both GPA and self-perceived employability, whereas GPA and self-perceived employability were unrelated to one another*. It is worth stating this explicitly, because a superficially similar claim — that procrastination bears on academic performance but not on employability — does not follow from these data: procrastination is reliably associated with both.

Procrastination predicted internal employability (b = −0.034, SE = 0.008, β = −.19, t(520) = −4.51, *p* < .001, R^2^ = .038), external employability (β = −.18, t(520) = −4.17, *p* < .001, R^2^ = .032) and the general score (β = −.22, t(520) = −5.20, *p* < .001, R^2^ = .050). Entering GPA alongside procrastination as a predictor of internal employability left the procrastination coefficient essentially unchanged (b = −0.040, t(448) = −4.84, *p* < .001) while GPA remained non-significant (b = −0.166, t(448) = −0.87, *p* = .39), confirming that the procrastination–employability association does not operate through academic attainment. Activity, the only temperament dimension associated with external employability, predicted internal employability (β = .19, t(520) = 4.31, *p* < .001, R^2^ = .034) and the general score (β = .19, *p* < .001).

Two incidental group differences are worth recording because they qualify the interpretation offered below. Women obtained higher grades than men (M = 4.19 vs 4.03; t(272) = 3.88, *p* < .001, d = 0.43) but reported *lower* internal employability (M = 6.91 vs 7.44; t(255) = −3.50, *p* < .001, d = −0.34). The two groups did not differ in procrastination (*p* = .43).

### Indirect associations between temperament and internal employability

The seven path models are reported in Table 4. In every model, temperament predicted procrastination (path a) and procrastination predicted internal employability while adjusting for temperament (path *b*, range −0.026 to −0.034, all *p* ≤ .001). The models accounted for between R^2^ = .009 (perseveration) and R^2^ = .100 (briskness) of the variance in procrastination, and between R^2^ = .038 (rhythmicity) and R^2^ = .089 (emotional reactivity) of the variance in internal employability.

**Table 4:** Regression-based path analysis: indirect association between each temperament dimension and self-perceived internal employability through procrastination.

Temperament dimension (X)	Effect on procrastination a (SE)	Effect of procrastination on internal employability b (SE)	Total effect on internal employability c (SE)	Direct effect c′ (SE)	Indirect effect ab [95% BCa CI]	ab (std.)	Pattern
Briskness	−0.410*** (0.054)	−0.028*** (0.008)	0.035*** (0.010)	0.023* (0.010)	0.012 [0.005, 0.020]	.051	Complementary
Perseveration	0.125* (0.057)	−0.032*** (0.008)	−0.025* (0.010)	−0.021* (0.010)	−0.004 [−0.010, −0.001]	−.018	Complementary
Sensory sensitivity	−0.217*** (0.061)	−0.031*** (0.008)	0.037*** (0.011)	0.030** (0.011)	0.007 [0.002, 0.014]	.027	Complementary
Emotional reactivity	0.220*** (0.045)	−0.026*** (0.008)	−0.048*** (0.008)	−0.043*** (0.008)	−0.006 [−0.011, −0.002]	−.030	Complementary
Endurance	−0.161*** (0.047)	−0.032*** (0.008)	0.019* (0.008)	0.014 (0.008)	0.005 [0.002, 0.011]	.027	Indirect only
Activity	−0.143** (0.052)	−0.030*** (0.008)	0.038*** (0.009)	0.034*** (0.009)	0.004 [0.001, 0.010]	.021	Complementary
Rhythmicity	−0.389*** (0.063)	−0.034*** (0.008)	0.011 (0.011)	−0.003 (0.012)	0.013 [0.007, 0.023]	.051	Indirect only

N = 522. Unstandardised ordinary least squares coefficients with standard errors in parentheses. *a* = effect of the temperament dimension on procrastination; *b* = effect of procrastination on internal employability, adjusting for the temperament dimension; *c* = total effect of the temperament dimension on internal employability; *c′* = direct effect of the temperament dimension on internal employability, adjusting for procrastination; *ab* = indirect effect with 95% bias-corrected and accelerated bootstrap confidence interval (5,000 resamples). *ab* (std.) = completely standardised indirect effect. “Pattern” follows the typology of Zhao, Lynch and Chen [66]: *complementary* = significant *ab* and *c′* with the same sign; *indirect only* = significant *ab* with non-significant *c′*. * *p* < .05, ** *p* < .01, *** *p* < .001.

The bias-corrected and accelerated 95% confidence interval for the indirect component excluded zero for all seven dimensions. Completely standardised indirect effects ranged from  .018 (perseveration) to  .051 (briskness and rhythmicity) in absolute value — small in Cohen’s conventional terms, and comparable to the indirect effects typically recovered in dispositional models of employability [67,68]. Five dimensions showed a *complementary* pattern, with the indirect and direct components sharing a sign. Two dimensions showed an *indirect-only* pattern: for endurance the direct association was non-significant (c′ = 0.014, p = .085) while the indirect component was reliable, and for rhythmicity neither the total (c = 0.011, *p* = .35) nor the direct association (c′ = −0.003, *p* = .81) reached significance although the indirect component was among the largest observed (*ab* = 0.013, 95% CI [0.007, 0.023]).

The rhythmicity result deserves particular attention. Rhythmicity has no detectable total association with internal employability, yet has a reliable indirect association through procrastination. Under the causal-steps criterion this pattern would be excluded from consideration; under the framework adopted here it is informative, indicating that a temperamental tendency toward regular, predictable patterning of daily activity is linked to employability appraisals *only* through its relation to delay behaviour. We note, however, that this pattern cannot be summarised as procrastination having “mediated” the temperament–employability relation: a confidence interval excluding zero establishes a non-zero product of coefficients, not a causal process.

### Supplementary analyses

Re-estimating all seven models with gender and age as covariates left every indirect component significant and changed no coefficient by more than  .002 (briskness *ab* = 0.013, 95% CI [0.006, 0.022]; perseveration −0.005 [−0.011, −0.001]; sensory sensitivity 0.007 [0.002, 0.013]; emotional reactivity −0.007 [−0.014, −0.003]; endurance 0.006 [0.002, 0.011]; activity 0.005 [0.001, 0.010]; rhythmicity 0.014 [0.007, 0.022]).

Replacing the outcome with external employability, and then with the general employability score, reproduced the pattern with slightly larger coefficients: all seven indirect components remained significant for both outcomes (external: standardised ab between  .017 and  .061; general score: between  .021 and  .069). This is consistent with the observation that procrastination is related to both facets of employability appraisal even though temperament is directly related mainly to the internal facet.

Harman’s single-factor test on the ten scale scores yielded a first unrotated component accounting for 24.5% of the variance, below the 50% threshold conventionally taken as indicative of severe common method bias. This test is a weak diagnostic [65] and does not rule out method variance; it is reported for transparency rather than as evidence of its absence.

Finally, principal component analysis of the seven FCB-TI(R) scale scores returned eigenvalues of 2.22, 1.37, 1.00, 0.83, 0.67, 0.59 and 0.33, with three components above unity and the first accounting for 31.7% of the variance. The rotated solution did not reproduce a clean reactivity–activity split. This analysis is the empirical basis for our decision not to specify a composite temperament predictor. A parallel analysis over 500 simulated data sets likewise retained two components rather than one, so no dominant general factor is present on either criterion.

Two further robustness checks are reported for completeness. Applying a Benjamini–Hochberg correction for multiple testing to the seven indirect effects on internal employability left five of them significant (q < .05), with perseveration (q = .075) and activity (q = .055) falling just outside the conventional threshold; no other conclusion is affected. Substituting Spearman rank correlations for Pearson correlations changed no coefficient in Table 3 by more than  .03, indicating that the associations do not depend on distributional assumptions

## Discussion

This study examined how the formal characteristics of behaviour relate to Polish first-year students’ confidence in their own employability, and whether procrastination is the behavioural channel through which that relation runs. Four findings warrant discussion: the differential contribution of individual temperament dimensions; the asymmetry between internal and external employability; the interpretive status of the indirect associations; and the dissociation between academic attainment and employability appraisal.

### Which temperament dimensions matter, and why the temporal ones matter most

Our results indicate that procrastination is negatively associated with activity and endurance, which comprise the energetic aspects of behaviour, consistent with accounts that position procrastination as a failure of self-regulation [31,69]. First-year undergraduates in particular struggle to manage their time effectively and tend to procrastinate because they overestimate their academic abilities [70]. Individuals who procrastinate show a lower capacity to respond adequately in situations demanding sustained or highly stimulating activity, and under intense external stimulation they tend to postpone performance. Our data also indicate that procrastinators react intensively to emotion-generating stimuli, reflected in high emotional reactivity and low endurance [71].

Comparing the dimensions directly — asking which aspect of temperament is most strongly implicated — yields a clear answer, and one that refines rather than confirms the standard account. The two largest associations with procrastination were briskness (r = −.32) and rhythmicity (r = −.26), both temporal characteristics, and both larger than the associations with the energetic characteristics of endurance (r = −.15) and activity (r = −.12). Emotional reactivity, an energetic characteristic with a strong affective component, was intermediate (r = .21). The same ordering held for the strength of the indirect components: briskness and rhythmicity produced the largest completely standardised indirect effects (.051 each), roughly twice those of activity (.021) and perseveration (.018).

This suggests that procrastination in this sample is bound up less with a deficit of energy or stamina than with the tempo and regularity of behaviour: a slower shift between activities and a lower rate of response (low briskness) and an irregular, unpredictable patterning of daily activity (low rhythmicity). The interpretation is strengthened by the fact that the same rank ordering – emotional reactivity and perseveration positive; briskness, endurance and rhythmicity negative – was obtained in an independent Polish adult sample using the same instrument [54]. That two independent samples drawn from different populations (students here; community adults there), measured with different procrastination instruments, converge on the same profile is stronger evidence than either study alone. Practically, it implies that interventions built on generic exhortation to “try harder” or on willpower are poorly targeted, whereas interventions that impose external temporal structure – fixed working rhythms, scheduled and segmented tasks, implementation intentions tied to times rather than to intentions – engage the dimensions that are actually implicated. This is consistent with evidence that emotion-regulation training specifically, rather than general self-control capacity, reduces procrastination [27], and with experience-sampling work showing that ambiguity in the task itself, not merely disposition, drives episodes of delay [72].

Perseveration deserves separate comment because its pattern is distinctive: it was the only dimension positively associated with procrastination that was also negatively associated with internal employability, and it had the weakest a-path in the set (R^2^ = .009). Perseveration denotes the tendency to continue or repeat behaviour after the stimulus that elicited it has ceased – dispositionally close to rumination. Its high correlation with emotional reactivity in our data (r = .62) suggests that the two operate as a single affective-repetitive complex, and that its relation to delay is more plausibly affective than temporal.

### Internal and external employability respond differently to disposition

The distinction between internal and external employability, though introduced early in the manuscript [17], deserves more analytical weight, because the two showed markedly different sensitivity to temperament. Internal employability (self-assessed skills and qualities) correlated significantly with five of seven temperament factors and with procrastination (*r* = −.22), whereas external employability (perceived opportunities in the labor market) correlated with only activity (*r* = .14), emotional reactivity (*r* = .10), and procrastination (*r* = −.16) with sensory sensitivity, endurance, perseverance, briskness, and rhythmicity all non-significant. This asymmetry makes conceptual sense: internal employability is a judgment about one’s own dispositions and capacities, so it is plausible that it would be more entangled with temperament – a similarly dispositional construct – than external employability, which depends more on perceptions of the labor market itself (economic conditions, sector demand, degree relevance) than on personal traits [50,51]. The pattern is what the enactive-mastery account predicts: an appraisal informed by one’s own task history should track dispositions that shape that history, whereas an appraisal about market conditions should not [57]. This pattern is consistent with prior work distinguishing internal employability as a more self-referential, psychologically-loaded construct compared to the more externally-anchored, context-dependent nature of perceived market opportunity [49]. It is instructive that procrastination is nevertheless related to *both* facets, and to a similar degree. If procrastination affected only the appraisal of one’s own competence, its association with the external facet should be negligible. That it is not suggests either that habitual delay colours labour-market appraisals through a general negative-affective route – consistent with the finding that procrastination predicts hopelessness in job-seeking [42] and subsequent mental-health difficulty [39] – or that students who delay simply gather less information about the labour market and therefore judge it less favourably. Our design cannot distinguish these accounts, and doing so would be a worthwhile target for future work.

Practically, this suggests that interventions targeting temperament-linked procrastination (e.g., time-management or self-regulation training^26^) may do more to shift students’ *confidence in their own qualifications* than their perception of the broader job market, which likely requires separate, structurally-oriented interventions (e.g., transparent labor-market information, employer engagement [73]).

Our study emphasizes the mediating role of procrastination in the relationship between temperament and internal employability (i.e., the self-perception of skills and qualities related to employability outcomes). A delayed task appears to have lower stimulative value. However, the more complex and difficult the activity, the higher the stimulation. Alternatively, an activity may include risk-taking or threatening actions; thus, individuals may procrastinate due to low levels of confidence in personal resources. This suggests a connection with the individual perspective of employability, which includes factors such as subject knowledge [23] and job experience, especially when reflected upon, and personal soft skills such as self-confidence, self-awareness, communication, efficacy beliefs, metacognition, and enterprise skills [49]. We are, however, deliberately cautious about the language used to describe this. The seven confidence intervals reported in Table 4 establish that the products of two regression coefficients are unlikely to be zero in a model that assumes the ordering temperament → procrastination → employability. They do not establish that ordering. With all three constructs measured at one occasion, the same covariance structure would be produced by a model in which low employability confidence encourages delay, or by a model in which an unmeasured third variable – trait self-efficacy, conscientiousness, or depressive symptomatology are the obvious candidates – generates all three. The developmental literature offers indirect support for our ordering, in that temperamental self-regulation predicts delay behaviour in pre-school children [56], long before employability appraisals exist; but this is an argument about plausibility, not identification. Recent longitudinal work using cross-lagged panel models illustrates both the feasibility and the necessity of the stronger design: reciprocal relations between academic self-concept and components of procrastination have been examined across a semester, revealing cross-lagged relations that no cross-sectional design could have detected [74].

One result carries a substantive message independent of causal interpretation. For rhythmicity, the total association with internal employability was indistinguishable from zero while the indirect component was among the largest observed. A researcher applying the causal-steps criterion would have concluded that rhythmicity is irrelevant to employability. The correct conclusion is that its relevance is entirely channelled through delay behaviour: temperamental irregularity is not itself demoralising, but it is reliably associated with postponement, and postponement is reliably associated with lower career confidence. This is a concrete illustration of why a significant total effect should not gate the examination of indirect effects [60.61].

### Academic attainment and employability confidence are dissociated

The most striking pattern in our data is that GPA showed no significant association with any of the three employability indices (|r| ≤ .02), even though procrastination was significantly associated with GPA (r = −.17), internal employability (r = −.19) and external employability (r = −.18). Procrastination therefore appears to bear on academic performance and on employability appraisal through separate channels, rather than academic performance acting as the pathway between them. The regression in which GPA and procrastination competed as predictors of internal employability makes the point directly: the procrastination coefficient was unaffected by the inclusion of GPA, and GPA contributed nothing. This is consistent with employability being conceptualised as a distinct, competency- and confidence-based construct rather than a proxy for academic performance [51], and reinforces prior calls to measure employability in its own right rather than inferring it from grades^9,11^.

The GPA correlations also repay closer inspection than they received in the previous version. Two temperament dimensions were associated with grades in the *opposite* direction to their association with employability confidence. Activity, the tendency to undertake behaviour of high stimulative value, was the strongest temperamental correlate of internal employability (r = .19) yet was negatively associated with GPA (r = −.17). Endurance showed the same reversal in weaker form (r = .10 with internal employability; r = −.09 with GPA). The most parsimonious reading is a time-allocation account: highly active students distribute effort across a wider portfolio of stimulating pursuits – paid work, societies, sport, social life -which builds the experiential base for career confidence while competing with study time. If so, the trait that best predicts a student’s belief in their own employability is mildly detrimental to the metric by which institutions most often judge them. We note that this reading is post hoc and requires direct testing with time-use data.

The incidental gender difference reinforces the dissociation from a different direction. Women in this sample achieved higher grades than men (d = 0.43) while reporting lower confidence in their own employability (d = −0.34), with no difference in procrastination. A gender gap in career self-appraisal that runs opposite to the gap in measured performance is well documented in the employability literature and cannot be explained by attainment; it points instead to appraisal processes, and it is a further reason not to treat grades as a readiness indicator.

Perseveration, by contrast, was not significantly associated with GPA (r = .08, *p* = .08), so no claim is made here about a link between grades and the tendency to repeat behaviour after the eliciting stimulus has ceased.

Looking at the positive outcomes in the context of global economic instability, the digitalisation of work and competitive graduate labour markets [4,20], students who perceive themselves as employable tend to exhibit goal-focused behaviour (activity), to respond quickly (briskness) and to organise their activity predictably (rhythmicity). In the context of students’ future employability, Sénécal and Guay [75] argued that high levels of procrastination toward job-seeking may increase hopelessness. Students with high hope have greater feelings of inspiration and confidence in goal pursuit than those with low hope [66,76] and students’ employment goals go hand in hand with academic achievement goals [77]. Procrastination may therefore place students at risk of a self-reinforcing sequence in which delay lowers both attainment and career confidence, and lowered confidence in turn makes future delay more likely — a possibility that the longitudinal health findings reported by Johansson and colleagues [39] make more than speculative, but which our cross-sectional design cannot confirm.

### Practical implications

Three major implications follow directly from the results reported above.

#### Do not use grades as a proxy for career readiness.

GPA was unrelated to every employability index in this sample, and the two constructs even ran in opposite directions with respect to gender and to the activity dimension of temperament. Academic advisers and career services should therefore track and support attainment and employability confidence as separate developmental outcomes: a student with a strong record may still report low confidence in their prospects, and vice versa. This distinction has particular force in the Polish higher education context, where competency-based employability provision remains less embedded than in comparator countries [47,50,78].

#### Target temporal structure rather than willpower.

Because the temperament dimensions most strongly implicated in procrastination were the temporal ones — briskness and rhythmicity — provision aimed at reducing delay is more likely to succeed if it externalises temporal structure than if it appeals to motivation. Concretely, this means scheduled and segmented work with fixed start times rather than deadline-anchored planning, tasks broken into units short enough to be initiated within a single work period, and explicit reduction of task ambiguity by instructors, which experience-sampling evidence identifies as a proximal trigger of delay episodes [72]. Since first-year students are both the group most susceptible to delay [38] and the group whose employability provision is typically deferred to the final year, entry-level placement of such provision follows.

#### Address the two facets of employability with different instruments.

Procrastination was related to both internal and external employability, but temperament was related almost exclusively to the internal facet. Psychological provision – self-regulation and emotion-regulation training [25,26] – can reasonably be expected to shift students’ confidence in their own qualifications. It should not be expected to shift their reading of the labour market, which calls for structural measures: transparent, field-specific labour-market information [73], employer engagement, and substantial work-based learning through internships or placements [4,48,67,79], which is well embedded in comparator European systems and remains the most direct means of altering perceptions of external opportunity.

A cautionary note is warranted on the well-being dimension. A tendency to postpone career decision-making may carry mental-health implications: procrastination toward job-seeking has been linked to hopelessness [42,75]and, prospectively, to mental-health problems, poor sleep, loneliness and economic difficulty [39,40]. Students who delay career decisions – for example by taking an unplanned gap year – may be avoiding an anticipated failure rather than making a considered choice [50] and this may reflect either a lifestyle preference [6] or genuine indecision about which positions to pursue [80]. Institutions should therefore treat marked career-decision delay as a possible well-being signal and not solely as a service-uptake problem [81].

### Study limitations and future research directions

The limitations of this study are substantial and constrain the inferences that can be drawn. First, the data are cross-sectional and correlational, and all three focal constructs were measured on a single occasion. The path models therefore estimate indirect *associations* under an assumed ordering; they cannot identify a mediating process, and they cannot exclude reverse ordering or unmeasured confounding [58,64]. Longitudinal designs with at least three waves, analysed with cross-lagged panel or random-intercept cross-lagged panel models, are required – the recent analysis of academic self-concept and procrastination across a semester provides a usable template [74] – and experimental manipulation of delay behaviour would be required to establish that reducing procrastination raises employability confidence.

Second, shared method variance may exist between the self-reported measures. Temperament, procrastination and employability were all assessed by self-report in a single sitting, so common method variance may inflate the associations among them. Harman’s single-factor test was reassuring (first component 24.5% of variance) but is a weak diagnostic [65]. The inclusion of GPA, drawn from institutional records rather than self-report, provides partial protection; notably, the procrastination–GPA association (r = −.17) is of similar magnitude to the wholly self-reported associations, which argues against method variance as the sole explanation. Future work should use a multi-trait multi-method design and, given documented inconsistency in the concurrent and predictive validity of self-report procrastination scales [82], should incorporate behavioural indices of delay such as submission timestamps or experience-sampling reports [72].

Third, no dedicated attention-check or instructed-response items were included in the questionnaire. Data quality was screened for completeness and invariant responding, but we cannot rule out inattentive responding, and we recommend that future administrations embed at least two instructed-response items.

Fourth, the sample was drawn from a single mid-sized Polish university and consisted of first-year students, 71.5% of them women. Generalisation to other institutional types, to later years of study, and to other national systems is not warranted without replication; cross-cultural work indicates that the dispositional correlates of perceived employability, while broadly robust, are not identical across contexts [52]. The predominance of women is also a constraint given the gender difference in employability appraisal reported above.

Finally, the model omits variables with a strong claim to inclusion. Self-efficacy, hope and grit have all been shown to carry dispositional effects on employability in Polish and international samples [45,53], and the environment of the task — its ambiguity, structure and deadline regime — has been shown to drive delay independently of disposition [72]. A model that includes both person and task-level predictors, ideally in a multilevel or experience-sampling design, would be a natural next step. We also intend to incorporate insights from recent Polish research on the impact of social class and parental influence on educational choices [83]. This study forms part of an ongoing programme of employability research that we aim to develop along these lines.

## Conclusions

Among Polish first-year undergraduates, the temporal characteristics of temperament understood as the pace and regularity of behaviour rather than its sheer energy – were the dimensions most strongly associated with procrastination, and procrastination was in turn associated with lower confidence in one’s own employability. For every temperament dimension, including one with no total association with employability at all, a reliable indirect component ran through procrastination. Because the design is cross-sectional, these results describe a coherent pattern of association rather than a demonstrated causal chain. Their practical significance lies elsewhere: academic attainment, the metric institutions most often use to judge readiness for work, was unrelated to students’ appraisal of their own employability, whereas a modifiable behaviour - delay – was related to both. Employability confidence and academic performance should be supported as distinct outcomes, and provision aimed at reducing delay should be built around temporal structure rather than exhortation.

## Supporting information

S1 File Authors’ Note. A note dedicated to the memory of Professor Andrew Rothwell, acknowledging his contribution to employability research and his mentorship of the authors’ research group.
(PDF)
