# Supplementary material for: The procrastination trap: How temperament undermines students’ career readiness in Poland
Source: PLoS One. 2026 Oct 16;21(10):e0358981. doi: 10.1371/journal.pone.0358981 (PMC13674774; doi:10.1371/journal.pone.0358981)
Supplement: Supplementary file 1 [file pone.0358981.s001.pdf]

S1. Authors' Note

This article is dedicated to the memory of Professor Andrew Rothwell, a distinguished scholar and leading expert in the field of employability. Professor Rothwell was not only an important voice in employability research, but also a generous mentor and an enduring source of inspiration to our research group. His intellectual contribution, kindness, and commitment to advancing the field have left a lasting mark on us and on the wider research community.

We publish this work in his honour, with deep gratitude for his mentorship and in recognition of his lasting contribution to employability research.
